# The prolongation effect of ilaprazole-based standard triple therapy for Helicobacter pylori: Erratum

**DOI:** 10.1097/MD.0000000000024431

**Published:** 2021-01-22

In the article, “The prolongation effect of ilaprazole-based standard triple therapy for Helicobacter pylori”,^[1]^ which appears in Volume 99, Issue 38 of *Medicine*, the conflict of interest statement, “The study was funded by Ilyang, which manufactures Ilaprazole”, was left out and has since been added to the article.
